# Präklinische Versorgung von anaphylaktischen Reaktionen durch die Dresdner Luft- und Bodenrettung: Analyse von Therapie und Outcome

**DOI:** 10.1007/s00106-024-01457-4

**Published:** 2024-04-09

**Authors:** Mandy Cuevas, Mark Frank, Wladimir Haacke, Theresa Lüdke

**Affiliations:** 1 Medizinische Fakultät und Universitätsklinikum Carl Gustav Carus, Klinik und Poliklinik für Hals‑, Nasen- und Ohrenheilkunde, Technische Universität Dresden, Fetscherstraße 74, 01307 Dresden, Deutschland; 2 Praxis für Hals‑, Nasen‑, Ohrenheilkunde und Allergologie, Freital, Deutschland; 3 Zentrale interdisziplinäre Notaufnahme, Städtisches Klinikum Dresden Friedrichstadt, Dresden, Deutschland; 4 DRF Stiftung Luftrettung gAG, Filderstadt, Deutschland; 5 Wissenschaftlicher Arbeitskreis der DRF Stiftung Luftrettung gemeinnützige AG, Filderstadt, Deutschland; 6 Brand- und Katastrophenschutzamt Dresden, Feuerwehr und Rettungsdienst, Dresden, Deutschland

**Keywords:** Immunsystemerkrankungen, Anaphylaxie, Allergische Reaktion vom Soforttyp, Notfallbehandlung, Adrenalin, Immune system diseases, Anaphylaxis, Immediate hypersensitivity, Emergency treatment, Adrenaline

## Abstract

**Hintergrund:**

Anaphylaxie kann lebensbedrohlich sein. Eine schnelle Diagnose und sofortige Therapie sind daher notwendig. Im Fall einer Anaphylaxie mit kardiovaskulärer und/oder respiratorischer Beteiligung wird die unmittelbare Verabreichung von Adrenalin i.m. (oder i.v. bei ausreichender medizinischer Erfahrung) in Leitlinien empfohlen. Bisherige Studien ergaben, dass bei Anaphylaxien häufig keine leitliniengerechte Therapie durchgeführt und insbesondere Adrenalin selten verabreicht wird.

**Fragestellung:**

Ziel dieser Arbeit war es, die Daten der Dresdner Luft- und Bodenrettung hinsichtlich der Therapie und des Outcomes von Patient/innen mit Anaphylaxien zu untersuchen. Zudem erfolgte eine Gegenüberstellung der Ergebnisse aus der Luft- und Bodenrettung. Der Fokus lag auf der Verabreichung von Adrenalin sowie auf dem Outcome.

**Material und Methoden:**

Retrospektiv wurden die Einsätze der Dresdner Bodenrettung (von 2012 bis 2016) sowie Luftrettung (von 2008 bis 2015), die aufgrund anaphylaktischer Reaktionen stattfanden, ausgewertet. Für alle Einsätze wurde der Schweregrad der Anaphylaxie, die verabreichte Notfallmedikation sowie die weitere Überwachung und der Ausgang untersucht.

**Ergebnisse:**

Für die Luftrettung wurden 152 Erwachsene/29 Kinder und für die Bodenrettung 1131 Erwachsene/223 Kinder ausgewertet. Erwachsene mit höhergradiger Anaphylaxie (Grad II–IV) erhielten in 19 % (Luftrettung) bzw. in 7 % (Bodenrettung) der Fälle Adrenalin. Bei den Kindern waren es 7 % (Luftrettung) bzw. 8 % (Bodenrettung) der Fälle. Die Therapie mit Antihistaminika und/oder Glukokortikoiden und/oder Adrenalin führte bei 96–98 % (Luftrettung) bzw. 72–77 % der Fälle (Bodenrettung) zu einer Verbesserung. Es wurde ein signifikanter Zusammenhang zwischen Verabreichen von Adrenalin und Verbesserung des Outcomes in der Kohorte der Bodenrettung gezeigt.

**Schlussfolgerung:**

In der Mehrheit der höhergradigen Anaphylaxien erfolgte trotz Leitlinienempfehlung keine Therapie mit Adrenalin. Der in dieser Studie gezeigte signifikante Einfluss von Adrenalin auf den Outcome unterstreicht den Handlungsbedarf zur Optimierung der Notfallbehandlung anaphylaktischer Reaktionen.

## Abstract (en)

**Background:**

Anaphylaxis can lead to a life-threatening situation. Therefore, rapid diagnosis and emergency treatment is indicated. Immediate treatment with intramuscular adrenaline (or intravascularly with sufficient medical expertise) in anaphylaxis with cardiovascular and/or respiratory symptoms is recommended by various guidelines. Previous studies have shown that anaphylaxis is often not treated according to guidelines and that adrenaline in particular is rarely administered.

**Objective:**

The aim of the study was to investigate the data on emergency treatment and outcomes of patients with anaphylaxis by the air rescue and ground-based emergency services Dresden. Moreover, the results of the air- and ground-based services were compared. The focus was on administration of adrenaline and outcomes.

**Materials and methods:**

Data from the ground-based emergency service Dresden (from 2012 to 2016) and the air rescue service Dresden (from 2008 to 2015) related to anaphylactic reactions were analyzed retrospectively. The severity of the anaphylaxis, the emergency medication administered, further monitoring, and outcomes were analyzed.

**Results:**

Data from 152 adults/29 children were analyzed for air rescue and 1131 adults/223 children for the ground-based emergency service. Adults with grade II–IV anaphylaxis received adrenaline in 19% (air rescue) and 7% (ground rescue) of cases. Children with grade II–IV anaphylaxis received adrenaline in 7% (air rescue) and 8% (ground rescue) of cases. Immediate drug therapy led to an improvement in general condition in 96–98% (air rescue) and 72–77% (ground-based emergency service) of cases. A significant correlation was found between the administration of adrenaline and the improvement in outcome in the ground-based emergency cohort.

**Conclusion:**

Guideline-compliant first-line therapy with adrenaline was not performed in the majority of the cases analyzed in this study. The significant effect of adrenaline on outcome demonstrated in this study emphases the need to improve the emergency treatment of anaphylactic reactions.

## Einteilung und Behandlung von anaphylaktischen Reaktionen

Anaphylaktische Reaktionen sind potenziell lebensbedrohliche, akut-systemische Reaktionen, welche durch eine allergische Reaktion des Soforttyps (Typ I) sowie des Immunkomplextyps (Typ III) ausgelöst werden. Charakteristisch setzen die Symptome einer Anaphylaxie plötzlich ein und können sich rapide und enorm verschlechtern. Anhand der Symptome kann die Anaphylaxie nach der Klassifikation von Ring und Messmer [1] in die Schweregrade I–IV eingeteilt werden (Tab. 1). Eine anaphylaktische Reaktion kann zu alleinigen Hauterscheinungen wie Erythem oder Juckreiz führen (Grad I). Liegen kardiovaskuläre, respiratorische und/oder gastrointestinale Symptome vor, handelt es sich je nach Ausprägung der Beschwerden um eine Anaphylaxie vom Grad II–III. Grad IV ist durch den Atem‑/Kreislaufstillstand definiert. Die Dynamik einer Anaphylaxie ist unvorhersehbar und sehr variabel. Auch bei leichtgradigen Erstsymptomen ist ein schnelles Voranschreiten der Symptomatik bis hin zum plötzlichen Atem‑/Kreislaufstillstand möglich [2–4]. Die Einteilung der Schweregrade dient daher lediglich zur Orientierung, da die Übergänge fließend sind. Selbst nach verabreichter Notfallmedikation und kompletter Remission der Symptome kann es zudem zu einer erneuten Verschlechterung der Beschwerden im Sinne eines biphasischen Verlaufs kommen. Daher ist eine 24-stündige Überwachung von Patient/innen mit anaphylaktischen Reaktionen empfohlen [5]. Die Evidenz für die Behandlung von Anaphylaxien ist bisher gering. Weltweit existieren mehrere Leitlinien mit teils verschiedenen Empfehlungen bezüglich der Diagnose und Therapie. Die Anaphylaxie-Leitlinie der Deutschen Arbeitsgemeinschaft der Wissenschaftlichen Medizinischen Fachgesellschaften e. V. (Erstveröffentlichung 2007, aktuelle Überarbeitung von 2021) empfiehlt eine sofortige medikamentöse Therapie der Anaphylaxie in Abhängigkeit vom vorliegenden Schweregrads [5]. Für Grad-I-Reaktionen ist die Therapie mit H1- und H2-Rezeptor-Antagonisten sowie Glukokortikoiden empfohlen. Dies gilt auch für Reaktionen vom Grad II–III mit alleinigen gastrointestinalen Beschwerden ohne kardiorespiratorischer Komponente. Bei höhergradiger Anaphylaxie (Grad II–III) mit Beteiligung des Atemwegs bzw. des Herz-Kreislauf-Systems ist laut Leitlinie die sofortige i.m.-Applikation von Adrenalin die medikamentöse Therapie der ersten Wahl. Bei ausreichender (notfall)medizinischer Erfahrung kann alternativ eine i.v.-Gabe erfolgen. Nach Stabilisierung des Allgemeinzustands sind anschließend ebenfalls Antihistaminika sowie Glukokortikoide angeraten. Ziel dieser Arbeit war es, die Daten der Dresdner Luft- und Bodenrettung hinsichtlich der Therapie und des Outcomes von Patient/innen mit Anaphylaxien zu untersuchen und gegenüberzustellen. Der Fokus lag hierbei besonders auf der Verteilung der Schweregrade sowie der Verabreichung von Adrenalin. Zudem wurde die Überwachung nach dem abgeschlossenen Noteinsatz analysiert.

**Tab. 1:** Schweregrade der Anaphylaxie nach der Klassifikation von Ring und Messmer [1]

Grad	Symptome der anaphylaktischen Reaktion			
	Haut	Gastrointestinal	Respiratorisch	Kardiovaskulär
*I*	Juckreiz, Urtikaria, Angioödem, „Flush“	–	–	–
*II*	Juckreiz, Urtikaria, Angioödem, „Flush“	Erbrechen, Krämpfe, Übelkeit	Heiserkeit, Dyspnoe, Rhinorrhö	Tachykardie, Hypotension, Arrhythmie
*III*	Juckreiz, Urtikaria, Angioödem, „Flush“	Erbrechen, Defäkation	Larynxödem, Bronchospasmus, Zyanose	Schock
*IV*	Juckreiz, Urtikaria, Angioödem, „Flush“	Erbrechen, Defäkation	Atemstillstand	Kreislaufstillstand

## Material und Methoden

Für den Zeitraum von 2008 bis 2015 wurden alle standardisierten Einsatzprotokolle der Dresdner Luftrettung ausgewertet, die für Notfalleinsätze bei anaphylaktischen Reaktionen angelegt wurden. Zusätzlich wurden von 2012 bis 2016 alle Protokolle der Dresdner Bodenrettung von Einsätzen mit Assoziation zu Anaphylaxien analysiert.

Einschlusskriterien waren: Kinder von 0–17 Jahren, Erwachsene ab 18 Jahren, Dokumentation einer vorhandenen anaphylaktischen Reaktion, vollständige Dokumentation hinsichtlich Alter, Geschlecht, Symptome und Behandlung. Ausgeschlossen wurden Protokolle, welche alleinige allergische Lokalreaktionen und somit keinen Hinweis auf eine Anaphylaxie aufwiesen. Einige Protokolle beinhalteten keine Angaben bezüglich des Auslösers, des Outcomes und der weiteren Überwachung. Diese Protokolle wurden dennoch in die Analyse einbezogen. Die fehlenden Angaben wurden als „keine Angabe im Protokoll“ charakterisiert.

Die statistische Auswertung erfolgte mittels IBM SPSS Statistics 29 (Fa. SPSS Inc., Chicago/IL, USA). Für die Daten der Bodenrettung wurde ein χ^2^-Test sowie die Spearman-Korrelation für die Analyse der Korrelation zwischen Adrenalintherapie und Outcome angewandt. Es wurde ein Signifikanzniveau von *p* < 0,05 festgelegt. Für die Daten der Luftrettung erfolgte aufgrund der geringen Fallzahlen sowie einer nahezu vollständigen Besserung des Allgemeinzustands unter erfolgter Therapie keine Korrelationsanalyse.

## Ergebnisse

### Verteilung des Schweregrads der Anaphylaxie

Die ausgewerteten Daten (Symptome, Therapie, Hospitalisierung und Outcome) der Luftrettung umfassen 152 Erwachsene und 29 Kinder. Die Kohorte der Bodenrettung umfasst Daten von 1131 Erwachsene und 223 Kinder mit anaphylaktischen Reaktionen.

Anhand der im Einsatzprotokoll angegebenen Symptome wurden für die Luft- sowie Bodenrettungseinsätze die Schweregrade der Anaphylaxie nach der Klassifikation von Ring und Messmer [1] bestimmt. Die Tab. 2 zeigt die Verteilung der Schweregrade I–IV für Erwachsene und Kinder in der Gruppe der Luftrettung und der Bodenrettung.

**Tab. 2:** Verteilung der Schweregrade der Anaphylaxie bei Kindern und Erwachsenen. Gegenüberstellung von Luft- und Bodenrettung

	Luftrettung (%)		Bodenrettung (%)	
Schweregrad der Anaphylaxie	Erwachsene (*n* = 152)	Kinder (*n* = 29)	Erwachsene (*n* = 1131)	Kinder (*n* = 223)
*I*	26	48	48	54
*II*	31	35	40	39
*III*	42	17	12	7
*IV*	1	0	0	0

### Medikamentöse Notfalltherapie der Anaphylaxie insgesamt

In Tab. 3 ist die Notfallmedikation aufgeführt, welche bei allen anaphylaktischen Reaktionen (Grad I–IV) verabreicht wurde. Auch hier erfolgte die Gegenüberstellung von Luft- und Bodenrettung getrennt für Kinder und Erwachsene. In der Luftrettung erhielten 2,5 % der Erwachsenen und 24 % der Kinder keinerlei Notfallmedikation. In der Bodenrettung erhielten 25 % der Erwachsenen und 39 % der Kinder keine medikamentöse Therapie. Des Weiteren wird gezeigt, wie viele Patient/innen H1-Rezeptor-Antagonisten, H2-Rezeptor-Antagonisten, Glukokortikoide sowie Adrenalin erhielten. In der Gruppe der Luftrettung wurde Adrenalin bei 15 % der Erwachsenen und bei 7 % der Kinder verabreicht. In der Bodenrettung erhielten 5 % der Erwachsenen und 5 % der Kinder mit einer Anaphylaxie Adrenalin.

**Tab. 3:** Verabreichte Notfallmedikation bei Kindern und Erwachsenen mit Anaphylaxie Grad I–IV in der Luft- und Bodenrettung

	Luftrettung (%)		Bodenrettung (%)	
	Erwachsene (*n* = 152)	Kinder (*n* = 29)	Erwachsene (*n* = 1131)	Kinder (*n* = 223)
*Keinerlei Medikation*	2	24	25	39
*H1-Rezeptor-Antagonist*	88	52	69	45
*H2-Rezeptor-Antagonist*	68	17	57	24
*Glukokortikoide*	84	45	68	51
*Adrenalin*	15	7	5	5

### Notfallmedikation bei höhergradiger Anaphylaxie

Betrachtet man nur die Fälle mit höhergradiger Anaphylaxie (Grad II–IV mit respiratorischer bzw. kardiovaskulärer Beteiligung) ergibt sich bei der verabreichten Notfallmedikation die Verteilung in Tab. 4, welche zeigt, dass 2 % (Luftrettung) bzw. 20 % (Bodenrettung) der Erwachsenen mit einer Anaphylaxie vom Grad II–IV keine medikamentöse Notfalltherapie erhielten. Bei den Kindern waren es 7 % in der Luft- und 37 % in der Bodenrettung. Erwachsene mit einer höhergradigen Anaphylaxie erhielten in der Luftrettung in 19 % und in der Bodenrettung in 7 % der Fälle Adrenalin. Bei den Kindern waren es 7 % (Luftrettung) bzw. 8 % (Bodenrettung).

**Tab. 4:** Verabreichte Notfallmedikation bei Kindern und Erwachsenen mit höhergradiger Anaphylaxie (Grad II–IV) in der Luft- und Bodenrettung

	Luftrettung (%)		Bodenrettung (%)	
	Erwachsene (*n* = 112)	Kinder (*n* = 15)	Erwachsene (*n* = 591)	Kinder (*n* = 102)
*Keinerlei Medikation*	2	7	20	37
*H1-Rezeptor-Antagonist*	88	73	74	51
*H2-Rezeptor-Antagonist*	71	33	62	32
*Glukokortikoide*	83	60	71	52
*Adrenalin*	19	7	7	8

### Überwachung bei Anaphylaxie insgesamt

Des Weiteren wurde ausgewertet, wie die weitere Überwachung der Patient/innen mit einer Anaphylaxie vom Grad I–IV nach Beendigen des Noteinsatzes erfolgte. Die Tab. 5 zeigt, dass in der Luftrettung 88 % der Erwachsenen und 83 % der Kinder nach dem Noteinsatz in ein Krankenhaus eingeliefert wurden. In der Bodenrettung waren es 60 % der Erwachsenen und 74 % der Kinder. Erwachsene mit Anaphylaxie wurden in der Luftrettung in 9 % und in der Bodenrettung in 26 % der Fälle in der Häuslichkeit belassen. In der Gruppe der Kinder waren es 17 % (Luftrettung) bzw. 16 % (Bodenrettung). Zudem wird in Tab. 5 die Verweigerung der Krankenhauseinlieferung durch die Patient/innen bzw. den Eltern aufgeführt. Keinerlei Informationen über die weitere Überwachung nach Beendigen des Noteinsatzes enthielten bei Kindern 9 % bzw. bei Erwachsenen 10 % der Protokolle der Bodenrettung 9 % bzw. bei Erwachsenen 10 % der Protokolle der Bodenrettung.

**Tab. 5:** Art der Überwachung nach dem Noteinsatz bei Kindern und Erwachsenen mit Anaphylaxie in der Luft- und Bodenrettung

	Luftrettung (%)		Bodenrettung (%)	
	Erwachsene (*n* = 152)	Kinder (*n* = 29)	Erwachsene (*n* = 1131)	Kinder (*n* = 223)
*Krankenhauseinlieferung*	88	83	60	74
*Belassen in der Häuslichkeit*	9	17	26	16
*Ablehnung der Krankenhauseinlieferung*	3	0	4	1
*Keine Angabe im Protokoll*	0	0	10	9

### Überwachung bei höhergradiger Anaphylaxie

Die Tab. 6 zeigt die erfolgte weitere Überwachung im Fall einer Anaphylaxie vom Grad II–IV. In 92 % (Erwachsene) bzw. 100 % (Kinder) der Fälle der Luftrettung erfolgte eine Krankenhauseinweisung. Bei der Bodenrettung waren es 66 % (Erwachsene) und 83 % (Kinder). In wenigen Fällen wurde die stationäre Überwachung durch die Patient/innen bzw. die Eltern verweigert. In 3 % (Kinder) bzw. 10 % (Erwachsene) aller Protokolle der Bodenrettung wurde keine Angabe bezüglich der weiteren Überwachung dokumentiert.

**Tab. 6:** Art der Überwachung der Kinder und Erwachsenen mit höhergradiger Anaphylaxie (Grad II–IV) in der Luft- und Bodenrettung

	Luftrettung (%)		Bodenrettung (%)	
	Erwachsene (*n* = 112)	Kinder (*n* = 15)	Erwachsene (*n* = 591)	Kinder (*n* = 102)
*Krankenhauseinlieferung*	92	100	66	83
*Belassen in der Häuslichkeit*	5	0	21	13
*Ablehnung der Krankenhauseinlieferung*	3	0	3	1
*Keine Angabe im Protokoll*	0	0	10	3

### Outcome der Patientenkohorten

Die Datenauswertung der Luftrettung ergab, dass 98 % der Erwachsenen eine Besserung unter der Therapie mit Antihistaminika und/oder Glukokortikoiden und/oder Adrenalin zeigten. Eine Verschlechterung war für einen Einzelfall dokumentiert. In der Gruppe der Kinder zeigte sich eine Verbesserung in 96 % der Fälle. Für 4 % wurden gleichbleibende Beschwerden trotz Therapieverabreichung beschrieben. Aufgrund dieser Verteilung sowie der geringen Fallgröße wurde für die Luftrettung keine Korrelationsanalyse durchgeführt.

Die Datenauswertung der Erwachsenen mit einer Anaphylaxie aus der Bodenrettung zeigte, dass es unter der Notfalltherapie mit Antihistaminika und/oder Glukokortikoiden und/oder Adrenalin in 77 % aller Fälle zu einer Verbesserung des Allgemeinzustands kam. In 18 % wurde der Zustand als konstant dokumentiert. Eine Verschlechterung zeigten 0,4 % aller Erwachsenen unter erfolgter Notfallmedikation; 4,6 % aller Protokolle enthielten keine Information bezüglich des Outcomes.

Bei den Kindern mit Anaphylaxie im Bereich der Bodenrettung wurde in 72 % eine Verbesserung dokumentiert; 25 % zeigten sich während der Therapie konstant. Eine Verschlechterung wurde in keinem Fall protokolliert; 3 % der Protokolle enthielten keinerlei Aussage über den Outcome.

Mittels χ^2^-Test sowie der Korrelation nach Spearman wurde für die Kohorte der Bodenrettung ein Zusammenhang zwischen der Therapie mit Adrenalin und einem verbesserten Outcome untersucht. In der Gruppe der Kinder besteht ein signifikanter Zusammenhang zwischen der Therapie mit Adrenalin und einem verbesserten Outcome (χ^2^ (1) = 3,71; *p* = 0,05; φ = 0,131; Spearman r = 0,131; *p* = 0,05). Die Auswertung der Erwachsenendaten ergab hierfür ebenfalls eine signifikante Korrelation (χ^2^ (1) = 13,32; *p* = 0,001; φ = 0,111; Spearman r = 0,111; *p* < 0,001).

## Diskussion

Bereits 2022 analysierten die Autoren den Datensatz der Luftrettung hinsichtlich epidemiologischer Daten, Auslöser, Therapie und Überwachung von anaphylaktischen Reaktionen [6]. Ziel dieser Arbeit war die Gegenüberstellung der Daten der Luftrettung und der Bodenrettung Dresden bezüglich der Schweregrade der Anaphylaxie, der medikamentösen Therapie, der Überwachung sowie des Outcomes.

### Verteilung der Schweregrade

Die Gegenüberstellung der Verteilung der Schweregrade der anaphylaktischen Reaktionen der Luft- und Bodenrettung zeigt, dass innerhalb der Kohorte der Luftrettung deutlich mehr höhergradige Anaphylaxien vorkommen. Dies könnte dadurch erklärbar sein, dass bei schwerwiegender Anaphylaxie und damit einhergehender Lebensbedrohung häufiger die Luftrettung durch die Rettungsleitstelle alarmiert wird.

Worm et al. [7] werteten 2014 insgesamt 4000 Fälle von Anaphylaxien aus. Hierbei zeigten Erwachsene und Kinder in etwa 5 % der Fälle eine Grad-I-Anaphylaxie, in 50–60 % eine Grad-II-Anaphylaxie und in 35–45 % eine Grad-III-Anaphylaxie. Eine Grad-IV-Anaphylaxie wurde bei 3 % der Erwachsenen und bei 0,9 % der Kinder festgestellt. Die Auswertung von Worm et al. umfasste Daten des Anaphylaxie-Registers [8], in welchem vorrangig höhergradige Anaphylaxien gemeldet werden. Daher sind Grad-I-Anaphylaxien im Anaphylaxie-Register unterrepräsentiert.

Die Einteilung des Schweregrads in dieser Studie erfolgte anhand der dokumentierten Symptome im Notfallprotokoll. Eventuell vorliegende unvollständige oder fehlerhafte Dokumentation kann einen Einfluss auf die Ergebnisse haben.

### Medikamentöse Notfalltherapie

Die Datenauswertung der Luftrettung ergab, dass 2,5 % der Erwachsenen und 24 % der Kinder keinerlei Akutmedikation trotz vorliegender Anaphylaxie erhielten. In der Gruppe der Bodenrettung waren es sogar 25 % der Erwachsenen und 39 % der Kinder. Ein Grund hierfür könnte sein, dass in der Luftrettung ausschließlich Fachärzte als Notärzte eingesetzt werden, die meist langjährige notfallmedizinische Erfahrung aufweisen. Studiendaten aus verschiedenen Ländern zeigen, dass im Fall einer Anaphylaxie häufig keine leitliniengerechte Therapie durchgeführt wird [9, 10]. Die Daten dieser Studie zeigten, dass Erwachsene deutlich häufiger H1-/H2-Rezeptor-Antagonisten und Glukokortikoide erhalten haben als Kinder mit Anaphylaxien. Die deutlich geringere Therapierate bei Kindern kann auf eine bereits in der Literatur beschriebene Unsicherheit in der Notfallbehandlung von Kindern zurückzuführen sein [11]. Insbesondere die adäquate Medikamentendosierung sowie das Atemwegsmanagement stelle für zahlreiche Notärzte eine Herausforderung, die mit einer Unsicherheit und Stress einherginge, dar.

Adrenalin ist als First-Line-Medikament in der Notfallbehandlung von Anaphylaxien mit respiratorischer und/oder kardiovaskulärer Beteiligung empfohlen, obwohl hierfür bisher keine prospektiven kontrollierten Studien vorliegen [5, 12, 13]. Eine Auswertung des Anaphylaxie-Registers über den Zeitraum von 2014–2016 ergab, dass Adrenalin nur in 10–20 % der Fälle einer höhergradigen Anaphylaxie verabreicht wurde [14]. Die vorliegende Datenauswertung ergab, dass in der Gruppe der Luftrettung lediglich 19 % der Erwachsenen und 7 % der Kinder trotz einer höhergradigen Anaphylaxie Adrenalin i.m. erhielten. In der Gruppe der Bodenrettung waren es nur 7 % der Erwachsenen und 8 % der Kinder.

Eine mögliche Erklärung für die seltene Verabreichung der Notfallmedikation und insbesondere von Adrenalin ist die unzureichende allergologische Ausbildung des notfallmedizinischen Personals in Bezug auf die Beurteilung und Behandlung von Anaphylaxien [15]. Ein weiterer Grund für die zurückhaltende Verwendung von Adrenalin könnte die Angst vor Nebenwirkungen sein. Bei vorbestehenden Herz-Kreislauf-Erkrankungen kann Adrenalin zu schwerwiegenden Komplikationen führen [16]. Die Datenauswertung zeigte jedoch in keinem Fall eine Verschlechterung des Patientenzustands nach erfolgter Therapie. Dies lässt schlussfolgern, dass durch die verabreichte Notfallmedikation keine negativen Nebenwirkungen hervorgerufen wurden.

### Überwachung

Anaphylaktische Reaktionen können in jedem Stadium der Symptome spontan abklingen. Aber auch ein Fortschreiten des Schweregrads trotz adäquater Therapie ist möglich [5]. Bei 5–20 % kann sich innerhalb von 6–24 h nach erfolgreicher Therapie ein protrahierter oder biphasischer Verlauf entwickeln [17]. Aufgrund der unklaren Dynamik und des unklaren Verlaufs wird im Fall einer höhergradigen Anaphylaxie eine stationäre Aufnahme zur 24-stündigen Beobachtung empfohlen [5]. Die Datenauswertung zeigte, dass 9 % der Erwachsenen und 17 % der Kinder entgegen der Empfehlung nach Beendigung des Noteinsatzes per Luftrettung in der Häuslichkeit belassen wurden. In der Gruppe der Bodenrettung waren es sogar 26 % der Erwachsenen und 16 % der Kinder. Betrachtet man nur die Anaphylaxien vom Grad II–IV wurden in der Luftrettung 5 % der Erwachsenen in der Häuslichkeit belassen. Alle Kinder mit Anaphylaxie vom Grad II–IV wurden in ein Krankenhaus eingewiesen. In der Bodenrettung wurden trotz höhergradiger Anaphylaxie 21 % der Erwachsenen und 13 % der Kinder nicht weiter medizinisch überwacht.

### Outcome

Für die Auswertung des Outcomes wurden nur die Daten von Patient/innen ausgewertet, welche medikamentös mit Antihistaminika und/oder Glukokortikoiden und/oder Adrenalin behandelt wurden. Die Datenauswertung der Luftrettung ergab, dass 98 % der Erwachsenen und 96 % der Kinder eine Verbesserung ihres Zustands zeigten. In der Gruppe der Bodenrettung wurde für 77 % der Erwachsenen eine Verbesserung des Allgemeinzustands protokolliert, für 0,4 % eine Verschlechterung. Bei den Kindern kam es in 72 % zur Verbesserung. Laut Protokollauswertung kam es in keinem Fall zu einer Verschlechterung. Der bessere Outcome in der Gruppe der Luftrettung könnte durch eine adäquatere Behandlung durch die meist erfahreneren Notärzte erklärt werden. Interessant wären weitere Untersuchungen, ob durch eine konsequentere Medikation eine weitere Verbesserung der Beschwerden möglich wäre.

Die Korrelationsanalyse in der Gruppe der Bodenrettung ergab einen signifikanten Zusammenhang zwischen der Therapie mit Adrenalin und einem verbesserten Outcome. In Betracht der bisherigen geringen Evidenz bzw. der unzureichenden Studienlage ist dies ein Nachweis von enormer Bedeutung. Die vorliegenden Daten unterstreichen die klare Empfehlung der Leitlinien zur sofortigen Verabreichung von Adrenalin i.m. bzw. i.v. (bei entsprechender Kenntnis und ausreichender Erfahrung) im Fall einer Anaphylaxie mit kardiorespiratorischer Beteiligung.

### Ausbildungsdefizit

Die Symptome einer Anaphylaxie sind äußerst vielfältig, die Prävalenz ist gering. Die Diagnose der Anaphylaxie basiert ausschließlich auf der klinischen Beurteilung. Anaphylaxie ist ein Notfall, der eine rasche Diagnose und eine sofortige Therapie erfordert. Untersuchungen zufolge ist medizinisches Personal nicht gut darauf vorbereitet, Anaphylaxien zu erkennen und zu behandeln [18, 19]. Notfallmediziner müssen besser geschult werden, eine Anaphylaxie zu erkennen und von anderen Diagnosen abzugrenzen [20]. Die Europäische Akademie für Allergologie und klinische Immunologie (EAACI) empfiehlt den Einsatz von Simulationstraining und visuellen Hinweisen, um das Erkennen und die Behandlung anaphylaktischer Reaktionen in Notfallsituationen durch medizinisches Fachpersonal zu verbessern [21].

## Fazit für die Praxis

- Die schnelle Diagnostik und Therapie der Anaphylaxie ist aufgrund der potenziellen Lebensbedrohung von immenser Bedeutung.
- Bei höhergradigen anaphylaktischen Reaktionen (ab Grad II) mit Beteiligung der Atemwege bzw. des kardiovaskulären Systems wird eine sofortige Therapie mit Adrenalin i.m. oder i.v. bei ausreichender medizinischer Erfahrung in Leitlinien empfohlen.
- Die erhobenen Daten belegen, dass in der Bodenrettung nur 7 % der Erwachsenen und 8 % der Kinder mit höhergradiger Anaphylaxie Adrenalin erhielten.
- In der Luftrettung waren es bei den Erwachsenen nur 19 % und bei den Kindern 7 %.
- Für die Kohorte der Bodenrettung wurde gezeigt, dass die Notfalltherapie mit Adrenalin zu einer signifikanten Verbesserung des Outcomes führt.
- Die präsentierten Daten ermöglichen eine evidenzbasierte Unterstützung für die Optimierung der Notfallbehandlung anaphylaktischer Reaktionen.
